# Tracking C–H bond activation for propane dehydrogenation over transition metal catalysts: work function shines†

**DOI:** 10.1039/d3sc01057k

**Published:** 2023-05-18

**Authors:** Xin Chang, Zhenpu Lu, Xianhui Wang, Zhi-Jian Zhao, Jinlong Gong

**Affiliations:** a Key Laboratory for Green Chemical Technology of Ministry of Education, School of Chemical Engineering and Technology, Tianjin University China zjzhao@tju.edu.cn jlgong@tju.edu.cn; b Collaborative Innovation Center of Chemical Science and Engineering Tianjin 300072 China; c Joint School of National University of Singapore and Tianjin University International Campus of Tianjin University, Binhai New City Fuzhou 350207 China

## Abstract

The activation of the C–H bond in heterogeneous catalysis plays a privileged role in converting light alkanes into commodity chemicals with a higher value. In contrast to traditional trial-and-error approaches, developing predictive descriptors *via* theoretical calculations can accelerate the process of catalyst design. Using density functional theory (DFT) calculations, this work describes tracking C–H bond activation of propane over transition metal catalysts, which is highly dependent on the electronic environment of catalytic sites. Furthermore, we reveal that the occupancy of the antibonding state for metal–adsorbate interaction is the key factor in determining the ability to activate the C–H bond. Among 10 frequently used electronic features, the work function (*W*) exhibits a strong negative correlation with C–H activation energies. We demonstrate that e^−*W*^ can effectively quantify the ability of C–H bond activation, surpassing the predictive capacity of the d-band center. The C–H activation temperatures of the synthesized catalysts also confirm the effectiveness of this descriptor. Apart from propane, e^−*W*^ applies to other reactants like methane.

## Abstract 2

This paper described a descriptor e^−*W*^ to quantify the ability of C–H bond activation over transition metal catalysts theoretically and experimentally. The descriptor provides a starting point to quickly determine whether a new material of interest can activate C–H bonds.

## Graphical abstract

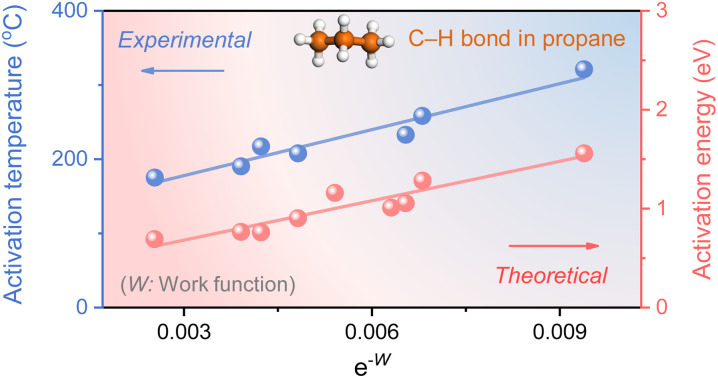

## Introduction

The thermodynamically stable nature of the C–H bond in alkanes, whose activation always accompanies high energy barriers, presents a formidable challenge for its conversion to value-added chemicals. With the exploitation of shale gas, light alkane dehydrogenation reaction has been even more important, for which catalysts play a privileged role.^1,2^ Specifically, propane dehydrogenation (PDH) to directly produce propylene, which is an on-purpose technology to satisfy the increasing demand for propylene, has drawn extensive attention in recent years.^2,3^ PDH consists of three steps: propane adsorption, surface reactions (first and second hydrogen abstraction) and propylene desorption.^4^ For the surface reactions, it is generally believed the first C–H activation step is the rate-determining one, whose activation energy can measure the PDH activity of catalysts and establish trends in C–H activation.^5–9^ Up to now, tremendous attempts have made to understand the dominating rules of C–H activation over catalytic surfaces, allowing for its prosperity over the past few decades.^1,10–12^ Other than the traditional approaches that rely on trial-and-error experiments, the concept of theory-guided catalysts design prevails. For instance, a thermodynamic descriptor, hydrogen affinity, can universally describe catalysts that follow the radical-like methane activation mechanism.^10^ As for the aspect of the electronic structure, d-band theory has been soundly established by Nørskov and co-workers,^13–17^ which profoundly addresses the adsorption behavior on catalytic surfaces of metal/alloy systems. In particular, the average energy of the d-states of a metal site (*i.e.*, the d-band center) correlates with the chemisorption strength of metal–adsorbate interaction. Generally, the surface sites with a higher-in-energy d-band center will bind adsorbates more strongly, and *vice versa*. Combined with scaling relationships, a quantitative understanding of trends in transition metal catalysis is developed, further constructing reactivity descriptors.^18^

Nevertheless, rapid prediction of catalytic properties over metal/alloy systems remains a challenge largely due to the limited applicability of the descriptor. The thermodynamic descriptors are mainly derived from the binding energy of key intermediates in a typical reaction (H,^19,20^ O,^21,22^ N,^23^*etc.*), which needs to investigate their adsorption behavior intensively. By contrast, the more easily acquired electronic descriptors are also inadequate for some cases. For example, the d-band center fails to describe the chemisorption of various adsorbates on Pd bimetallic alloys, calling for considering the effect of the d-band shape.^17^ In metal/alloy systems, the d-band center tuned by the surface strain also shows independent scaling relations with the reactivity based on the types of active sites.^24^ Moreover, for various transition metal catalysts, the d-band center can be less predictive, while the binding energy better describes the catalytic activity.^25^ Therefore, seeking more applicable descriptors at an electronic level is still a fascinating topic.

Herein, a systematic study on C–H bond activation is conducted, starting from the commonly used active metals M (M = Pt, Pd, and Ni). Typically, Pt has long been known as a highly active catalyst for alkane dehydrogenation because of its affinity for paraffinic C–H bonds, and a Pt-based catalyst is industrially and commercially used in Oleflex for PDH.^2,3^ Pd and Ni, especially the non-noble-metal Ni, are always used as an alternative to Pt to drive C–H bond activation.^26–29^ In the meantime, M-based alloys with alloy element Zn are investigated, considering that Zn can enable wide electronic state tuning of active sites.^28,30–34^ Through exploring the geometries of transition states and the electronic properties of active sites, we take advantage of one electronic feature, the work function (*W*), to develop a simple descriptor called e^−*W*^, whose effectiveness is validated by the C–H activation temperature of the synthesized catalysts. For the C–H bond activation of propane or methane, e^−*W*^ exhibits much better predictive capacity than the d-band center over 20 types of Pt-based alloys (alloying elements = 3d, 4d, and 5d metals). When transition metals in groups 8, 9, and 10 (Fe, Ru, Os, Co, Rh, Ir, Ni, Pd, and Pt) serve as active sites, their ability to activate the C–H bond in propane is remarkably correlated linearly with e^−*W*^, providing that active sites are within the same group.

## Results and discussion

**Fig. 1:**
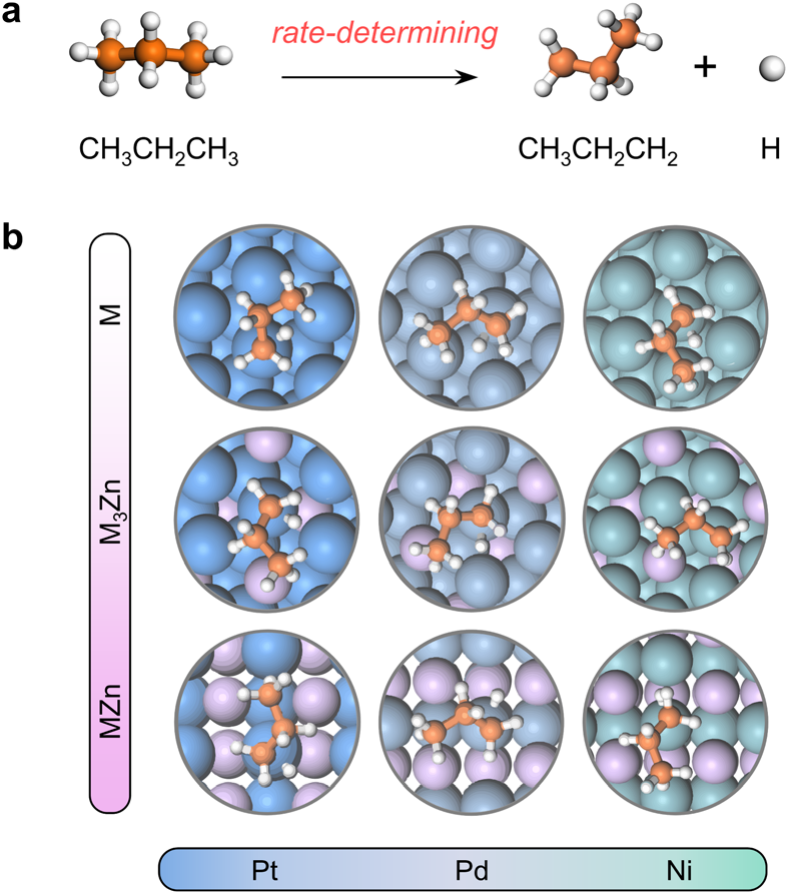
(a) The first C–H activation step of PDH reaction. (b) Geometries of the transition states for the first dehydrogenation step. Color: Pt-blue, Pd-light steel blue, Ni-cyan, C-orange, and H-white.

**Fig. 2:**
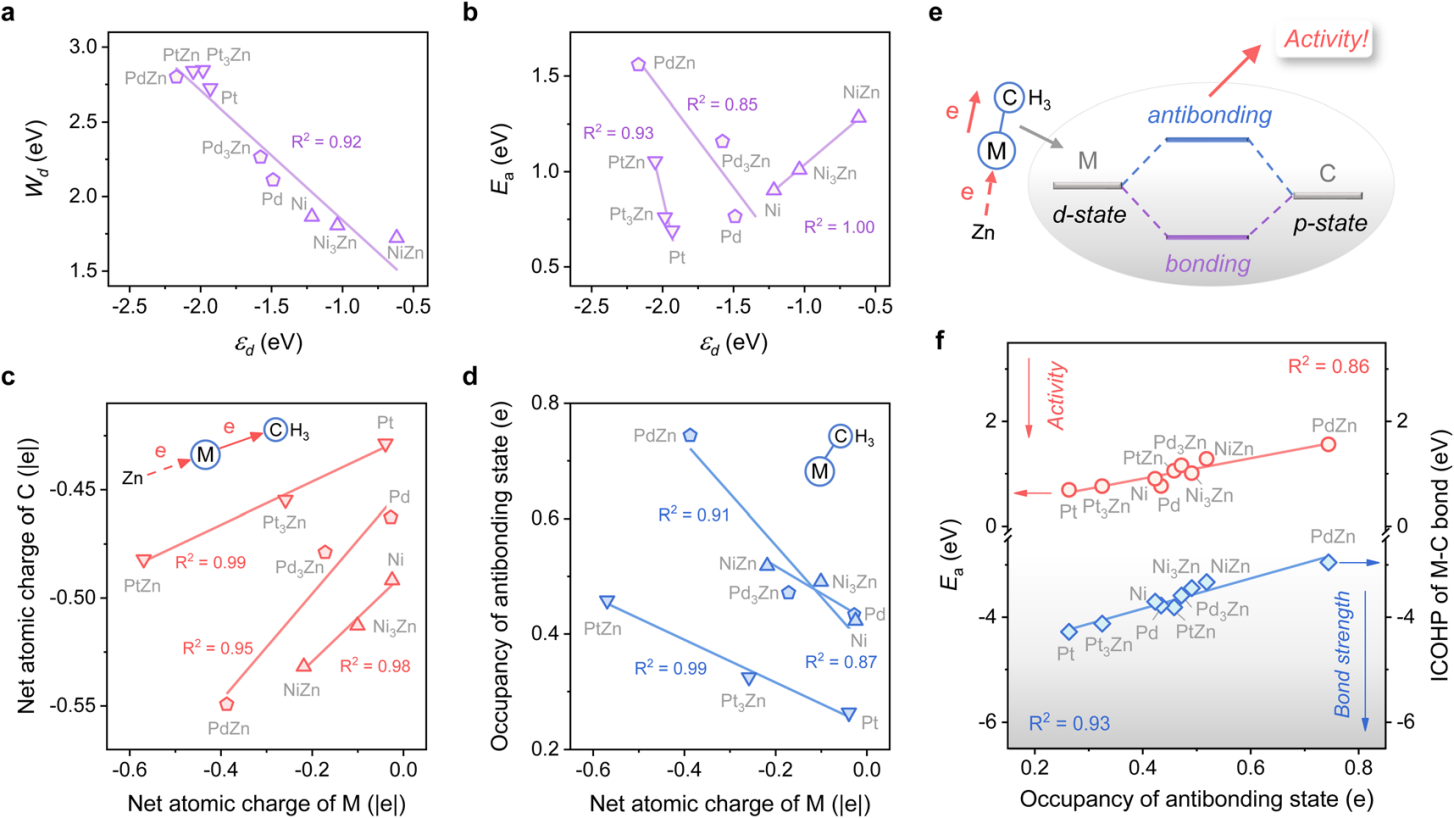
(a) Plot of the d-band width (*W*_d_) against the d-band center (*ε*_d_) of M. (b) Plot of the activation energy (*E*_a_) for the first dehydrogenation step against the d-band center (*ε*_d_) of M. (c) Plot of the net atomic charge of C against that of M. (d) Plot of the occupancy of the antibonding state of the M–C bond against the net atomic charge of M. (e) Schematic of the electronic interaction between the metal and adsorbate. (f) Plots of ICOHP of the M–C bond and the activation energy (*E*_a_) for the first dehydrogenation step against the occupancy of the antibonding state.

The close-packed surface (111) of Pt, Pd, Ni, Pt_3_Zn, Pd_3_Zn, and Ni_3_Zn with a face-centered cubic phase and (110) of PtZn, PdZn, and NiZn with a body-centered tetragonal phase were chosen for calculation. To simplify, M, M_3_Zn, and MZn are collectively called the M-class. Fig. 1b shows that the structure of transition states for the rate-determining step (Fig. 1a) are similar for all three classes, both with propyl and hydrogen contacting one active M atom, which indicates that the activity of propane dehydrogenation is mostly dependent on the electronic environment of catalytic surfaces. We first correlate the catalytic performance to the electronic density of states (DOS). Note that the d-band center (*ε*_d_) of various M becomes more negative as the d-band width (*W*_d_) is broader (Fig. 2a and S1†), in accordance with the perception of the d-band model.^15^

However, for the activation of the C–H bond, *ε*_d_ exhibits disparate trends over various types of metal surfaces. For the Pt-class and Pd-class, *ε*_d_ becomes more negative when the content of Zn increases, which is in contrast to the Ni-class (Fig. 2b and S1†). As illustrated by the d-band model,^13–15^ a metal site with a higher (lower) *ε*_d_ will exhibit stronger (weaker) adsorption strength; then the stronger (weaker) adsorbate–metal interaction will bring about enhanced (decreased) activity according to the Brønsted–Evans–Polanyi (BEP) relationship.^35^ This abnormal phenomenon indicates the restriction of the d-band model in describing the activity among various active metals, which hinders the establishment of a universal principle for the catalyst design. Additionally, we expect that the up-shift of *ε*_d_ from Ni to NiZn for the Ni-class can be mainly due to the narrower d-band, and the occupancy of the antibonding state for metal–adsorbate interaction still increases, contributing to decreased activity.

To further uncover why the activity changes, the electron density of the metal site and its influence on the electronic interaction of metal–adsorbate are both investigated using methyl^36^ as a detector. Fig. 2c shows that a more negative net atomic charge of M gives a higher electron density of C atoms when the adsorbate come into contact with the metal site. Moreover, the charge of M is influenced by the electronegativity difference between M and Zn, along with the ratio of Zn (Fig. S2†). As the M–C bond forms, a more negative atomic charge of M leads to a higher occupancy of the antibonding state (Fig. 2d). It is mainly due to the coupling of the d-state of metal and p-state of C, which is revealed by chemical bonding analyses with the Periodic Natural Bond Orbital (NBO) approach^37^ (Fig. 2e and Table S1†). The result of the integrated crystal orbital Hamilton population (ICOHP) also indicates that the occupancy of the antibonding state is the key factor in determining the M–C bond strength and then the dehydrogenation activity of diverse active metals (Fig. 2f). When the antibonding state increases, the activity drops off, consistent with our expectation above. In this regard, a universal design principle for C–H activation among various metals seems to be accessible.

**Fig. 3:**
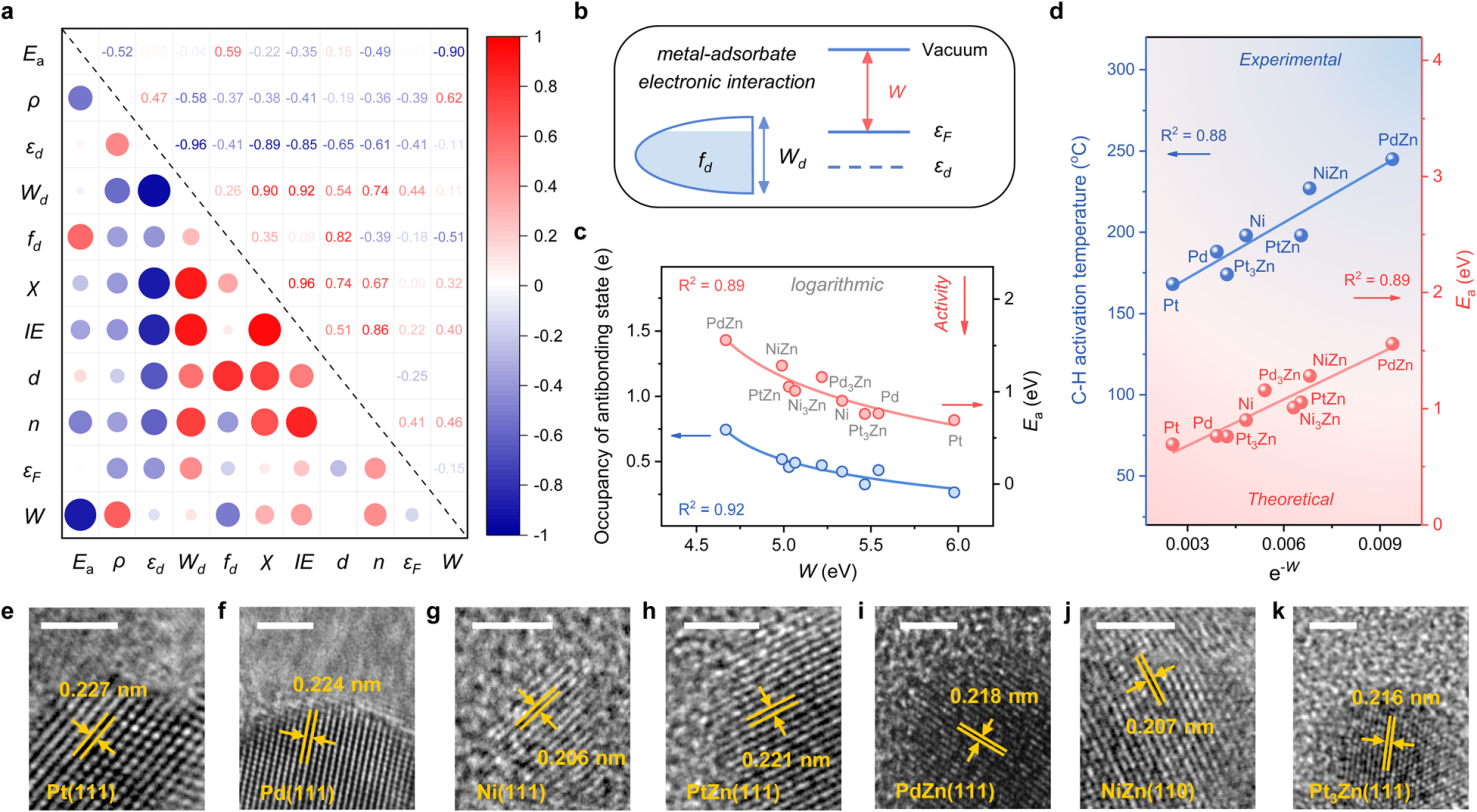
(a) Pairwise Pearson correlations between the electronic features and *E*_a_ over M, M_3_Zn, and MZn surfaces (M = Pt, Pd, and Ni). (b) Schematic of the d-band model and work function (*W*), which can contribute to the interaction between the metal and adsorbate. (c) Plots of the occupancy of the antibonding state of the M–C bond and the activation energy (*E*_a_) for the first dehydrogenation step against *W*. (d) Plots of the C–H activation temperature of the synthesized catalysts and the activation energy (*E*_a_) for the first dehydrogenation step against e^−*W*^. HRTEM images of (e) 2% Pt/SiO_2_, (f) 2% Pd/SiO_2_, (g) 2% Ni/SiO_2_, (h) 2% PtZn/SiO_2_, (i) 2% PdZn/SiO_2_, (j) 2% NiZn/SiO_2_, and (k) 2% Pt_3_Zn/SiO_2_. The scale bar is 2 nm.

Considering the complexity of calculating the occupancy of the antibonding state for the M–C bond, which requires evaluating the bond formation, finding a descriptive method merely based on the electronic feature is more appealing. Therefore, we investigate 10 frequently used electronic features of both the metal site and the surface (Table 1). Notably, *ε*_d_ exhibits a relatively small absolute Pearson correlation coefficient with *E*_a_, showing that this feature is uncorrelated with *E*_a_ (Fig. 3a). By contrast, the electron density (*ρ*) and d-band filling (*f*_d_) that describe the electronic environment of the metal site better correlate with *E*_a_. Since the C–H bond activation shows reliance on the properties of metal sites, it has pushed forward site-based characters to be used in previous research to describe the trend of C–H activation, which includes the adsorption strength of hydrogen over catalytic sites,^10^ the d-band center of metal sites,^7^*etc.*

**Table 1:** Electronic features and their notations

Features	Notation
Electron density	*ρ*
d-Band center	*ε*_d_
d-Band width	*W*_d_
d-Band filling	*f*_d_
Pauling electronegativity	*X*
Ionization energy	IE
Number of outer d electrons	d
Number of electron shells	*n*
Energy of the Fermi level	*ε*_F_
Work function	*W*

Given the above discussions established, we are surprised to find that the work function (*W*), a surface property, strongly correlates with *E*_a_. Specifically, *W* represents the ability of metal surfaces to exchange electrons with the environment in chemical processes,^38^ and a larger *W* will signify more difficult electron transfer (Fig. 3b). For the electron-donating intermediate (*e.g.* C_*x*_H_*y*_) that arises during the dehydrogenation reaction, its adsorption will be repelled by the surface site when the site is inclined to donate electrons. Thus, the larger the work function, the better the activity, as indicated by the negative correlation between *W* and *E*_a_ (Fig. 3a).

While disclosing the strongly correlated relationship, we notice that a logarithmic function can provide a better correlation between *W* and *E*_a_, along with the occupancy of the antibonding state (Fig. 3c and S3†). It means that *E*_a_ would not decrease all the time when *W* continues to increase. A physics intuition can tell that *E*_a_ is always larger than 0, indicating that the logarithmic correlation is more appropriate. Consequently, e^−*W*^ is used to describe the ability of C–H bond activation among different active metal sites, which shows a high predictive capacity for C–H bond activation (Fig. 3d). For more convincing, the rate-determining C–H activation step of the β-type PDH reaction is also calculated (Fig. S4†), which has a similar trend to the α-type one (Fig. 1 and 3d).

To validate the availability of this descriptor, SiO_2_-supported catalysts were synthesized. A set of diffraction peaks correspond well with the standard powder X-ray diffraction (XRD) patterns for the samples (Fig. S5†), which include Pt (PDF #70-2431), Pd (PDF #88-2335), Ni (PDF #70-1849), PtZn (PDF #06-0604), PdZn (PDF #06-0620), NiZn (PDF #65-3203) and Pt_3_Zn (PDF #65-3257). Herein, Pd_3_Zn and Ni_3_Zn are not included due to the unsteadiness of the structure. High-resolution transmission electron microscopy (HRTEM) images that measure the lattice spacings further reveal the successful synthesis of catalysts (Fig. 3e–k). The binding energy of M in the X-ray photoelectron spectroscopy (XPS) spectrum also indicates the electron transfer from Zn to M, consistent with our theoretical results (Fig. S6†). During temperature-programmed surface reaction (TPSR) analysis, propane–deuterium isotope scrambling (P–D scrambling) was used to compare the intrinsic ability of catalysts to activate the C–H bond of propane.^5^ The C–H activation starts at about 168 °C on Pt/SiO_2_, 188 °C on Pd/SiO_2_, 198 °C on Ni/SiO_2_, 174 °C on Pt_3_Zn/SiO_2_, 198 °C on PtZn/SiO_2_, 245 °C on PdZn/SiO_2_, and 227 °C on NiZn/SiO_2_, respectively (Fig. S7†). The activation temperatures show a nice linear relationship with e^−*W*^, underscoring the effectiveness of this descriptor (Fig. 3d).

**Fig. 4:**
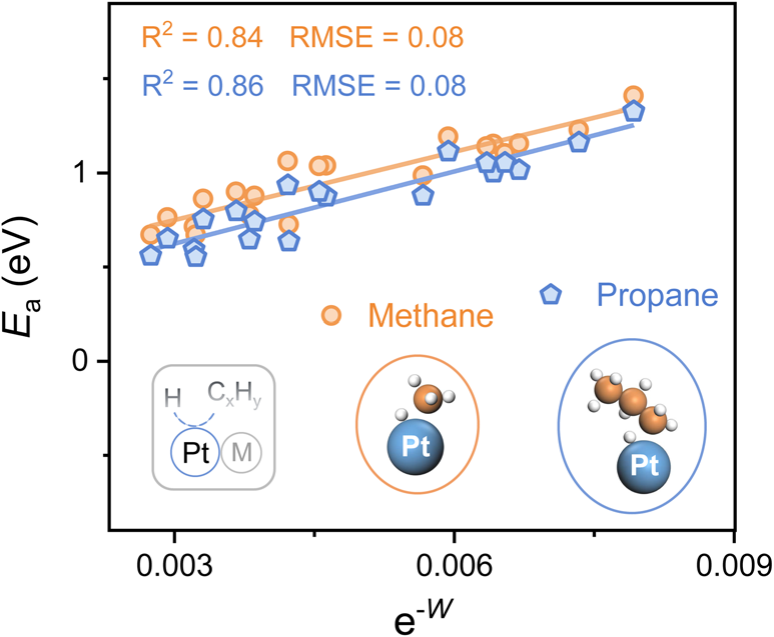
C–H bond activation for different reactants (methane and propane) over 20 types of PtM alloys. The alloying elements are Cr, Mn, Fe, Co, Ni, Cu, Zn, Nb, Mo, Tc, Ru, Rh, Pd, Ag, Cd, W, Re, Os, Ir, and Au. Plots of the activation energy (*E*_a_) for the first dehydrogenation step of propane and methane against e^−*W*^. The insets show the geometries of transition states. Color: Pt-blue, C-orange, and H-white.

To explore the applicability of e^−*W*^ for different reactants as well as various catalytic microenvironments, the C–H bond activation of propane and methane over 20 types of Pt-based alloys (alloying elements = 3d, 4d, and 5d metals) is investigated, whose geometries of the transition state for both propane and methane dehydrogenation are similar (Fig. 4). A universal scaling relationship exists for these reactants, and the propane activation line lies below and parallels the line of methane, as propane manifests lower bond dissociation energy of the C–H bond in contrast to methane. Moreover, the errors for the scaling relationships, evaluated from the Root Mean Square Error (RMSE), are lower than 0.1 eV, which is comparable to the classic accuracy of DFT adsorption energies.^39,40^

Hence, this single electronic descriptor, e^−*W*^, provides a promising approach to avoid the computationally expensive calculations of transition states for various C–H bond activation reactions over diverse catalytic surfaces, and accelerate the process of catalyst design. It's worth noting that the d-band center almost loses the predictive capacity of C–H bond activation for alloys with disparate microenvironments, taking account of the poor linear relationship (*R*^2^ < 0.3) (Fig. S8†). Despite its efficiency, *W*, one kind of surface property, is restricted in describing the C–H bond activation among a larger range of active metals. For instance, when metals in groups 8, 9, and 10 (Fe, Ru, Os, Co, Rh, Ir, Ni, Pd, and Pt) serve as active sites, the activity of the elements within the same group shows a linear relationship with e^−*W*^ while the slope increases from group 8 to 10 (Fig. S9†). It is easy to envision that the C–H bond activation over metal and alloy surfaces also relies on the active sites, whose intrinsic properties may be similar within the same group, further making the activation highly dependent on the surface electronic properties. Therefore, to establish a universal descriptor of activating the C–H bond over metal and alloy systems, both the intrinsic features of active sites and the properties of catalytic surfaces should be carefully investigated.

## Conclusions

In summary, the electronic regulation of metal sites alters metal–adsorbate interactions, whose occupancy of the antibonding state is the dominating factor in C–H bond activation, as revealed by electronic structure calculations and chemical bonding analyses. Moreover, among the easily accessible electronic features of metal sites and surfaces, the work function shines. Unlike the usual linear relationship, the work function shows a logarithmic correlation with the ability of C–H bond activation. Thus, a descriptor, e^−*W*^, is developed, which exhibits benign predictive capacity theoretically and experimentally. It can also extend to various alloys with disparate microenvironments and other reactants like methane. This work provides a starting point and a basis to quickly determine whether a new material of interest can activate the C–H bond successfully, calling for more efforts to find a universal descriptor.

## Data availability

The data that support the findings of this study are available within the article and its ESI,† or from the corresponding author on reasonable request.

## Author contributions

Xin Chang (conceptualization; methodology; investigation; visualization; formal analysis; validation; writing – original draft); Zhenpu Lu (investigation; formal analysis; writing); Xianhui Wang (investigation; formal analysis; writing); Zhi-Jian Zhao (supervision; writing – review & editing; resources and funding acquisition); Jinlong Gong (supervision; writing – review & editing; resources and funding acquisition).

## Conflicts of interest

There are no conflicts to declare.

## Supplementary Material

SC-014-D3SC01057K-s001
