# Supplementary material for: Tracking C–H bond activation for propane dehydrogenation over transition metal catalysts: work function shines
Source: Chem Sci. 2023 May 18;14(23):6414–9. doi: 10.1039/d3sc01057k (PMC10266452; doi:10.1039/d3sc01057k)
Supplement: SC-014-D3SC01057K-s001 [file SC-014-D3SC01057K-s001.pdf]

Electronic Supplementary Material (ESI) for Chemical Science.
This journal is © The Royal Society of Chemistry 2023

Supporting Information

Tracking C–H Bond Activation for Propane Dehydrogenation over Transition Metal Catalysts: Work Function Shines

Xin Chang,[†] Zhenpu Lu,[†] Xianhui Wang,[†] Zhi-Jian Zhao[*,†,‡] and Jinlong Gong[*,†,‡]

Table of Contents

Computational and experimental methods S3

Supplementary Figures...S4

Supplementary Table S16

References...S17

Computational details: The calculations were performed with the Vienna Ab initio Simulation Package (VASP)[1] using the projector augmented wave (PAW) method[2] and the generalized gradient approximation (GGA)[3] in the form of the Bayesian error estimation functional with van der Waals corrections (BEEF-vdW).[4] The planewave basis sets were converged at a kinetic energy cutoff of 400 eV for each slab model, of which the bottom two layers were fixed. The close-packed surfaces are chosen for calculation. For magnetic elements or alloys (including elements Ni, Fe, Co, Mn, Cr), spin-polarized DFT calculations were specially employed. An atomic force convergence criterion of 0.02 eV/Å was used to identify optimized geometries. For Pt(111), Pd(111), Ni(111), Pt_3Zn(111), Pd_3Zn(111), Ni_3Zn(111), (4×4) supercell with four layers was used, along with (3×3×1) k-point grid. For PtZn, PdZn, and NiZn alloys, the close-packed surface (110) was used. (2×2) supercell with five layers was applied, along with (3×3×1) k-point grid. 20 types of metals are used as alloy elements, which are Cr, Mn, Fe, Co, Ni, Cu, Zn, Nb, Mo, Tc, Ru, Rh, Pd, Ag, Cd, W, Re, Os, Ir, Au. Atomic charges were computed using Bader charge analysis.[5] Transition states (TSs) were determined with the climbing-image nudged elastic band (CI-NEB) method and the dimer method.[6, 7] Crystal orbital Hamilton population (COHP) analysis was performed with the LOBSTER 3.2.0 package.[8, 9]

Catalyst preparation: All the catalysts were prepared by the incipient wetness impregnation method using H_2O as the solvent. Amorphous SiO_2 (Alfa Aesar, 350-410 m^2/g, 325 mesh) was used as support. $H_2PtCl_6{\cdot}6H_2O$ (Chemart (Tianjin) Chemical Technology Co., Ltd, 99.9%), $PdCl_2$(J&K Scientific, 99.9%) $Ni(NO_3)_2{\cdot}6H_2O$(Aladdin (China) Chemical Co., Ltd, 99.0%) and/or $Zn(NO_3)_2{\cdot}6H_2O$ (Aladdin (China) Chemical Co., Ltd, 98.0%) was used as precursors, respectively. After impregnation, the catalysts were placed statically overnight and dried at 353 K for 12 h and then calcined at 873 K under 10% H_2-Ar atmosphere for 1 h. The metal loading is based on the weight ratio between metal and SiO_2, named as Pt/SiO_2, Pd/SiO_2, Ni/SiO_2, PtZn/SiO_2, PdZn/SiO_2 NiZn/SiO_2 and Pt_3Zn/SiO_2 with 2 wt % feeding of Pt, Pd or Ni.

The XRD measurements were performed on a Bruker D8 diffractometer operating at 200 mA and 40 kV, which employs the graphite filtered Cu Ka as the radiation source. The data points were collected by step scanning with a rate of 6 min^{-1}.

The XPS measurements were performed in ThermoFischer ESCALAB 250Xi photoelectron spectrometer.

The TEM images were taken using a JEOL JEM 2100 F system at an accelerating voltage of 200 kV equipped with a field emission gun.

TPSR of P–D scrambling was measured on the Micromeritics Auto Chem II 2920 chemisorption apparatus and the output products were analyzed on Hiden QIC-20 mass spectrometer (C_3H_7D, m/e equals to 30). Typically, 100 mg of the as-prepared catalysts were pre-reduced in U-type reactor at 873 K for 1 hours under 10 vol% H_2 in N_2 flow (20 mL/min) and cooled to 353 K. After the Ar purging in a flow rate of 20 mL/min, the samples were heated to 873 K at a rate of 10 K/min in a mixture of 5 vol% C_3H_8-5 vol% D_2 in He (20 mL/min).

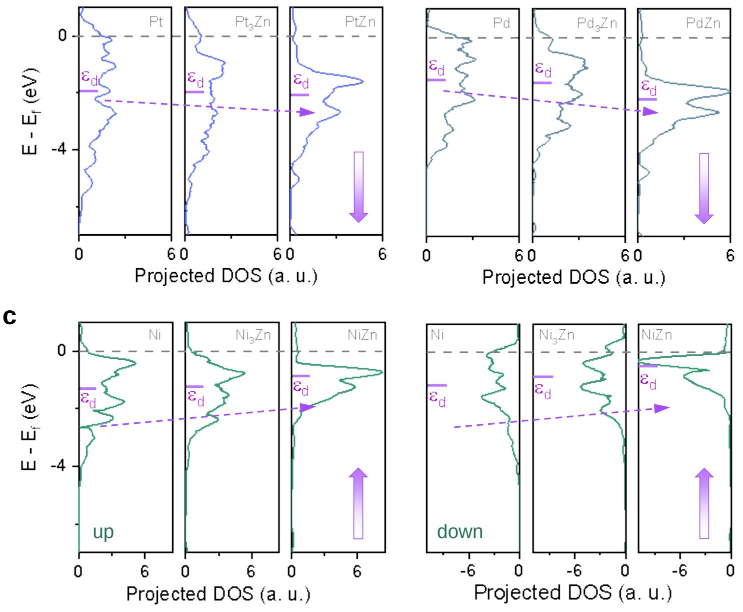

Figure S1. (a) DOS projected onto the *d*-bands of surface Pt atoms on the Pt, Pt_3Zn and PtZn surfaces. (b) DOS projected onto the *d*-bands of surface Pd atoms on the Pd, Pd_3Zn and PdZn surfaces. (c) DOS projected onto the *d*-bands of surface Ni atoms on the Ni, Ni_3Zn and NiZn surfaces. E_f is the fermi level and ε_d is the *d*-band center.

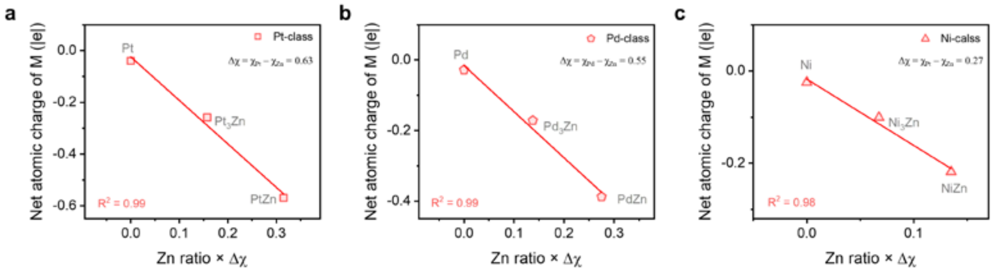

Figure S2. Plots of the net atomic charge of M against the product of Zn ratio in an alloy and the electronegativity difference. (a) Pt-class. (b) Pd-class. (c) Ni-class. For M, Zn ratio is 0; for M_3Zn, Zn ratio is 0.25; for MZn, Zn ratio is 0.5.

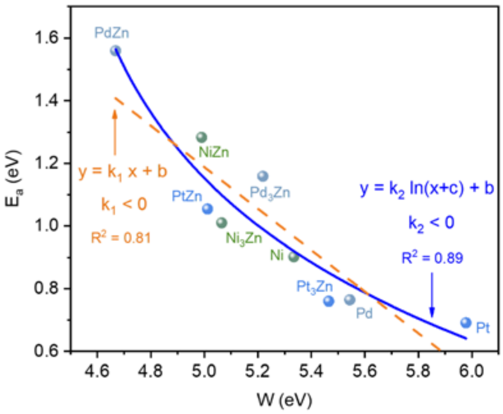

Figure S3. Plot of the activation energy (E_a) for first dehydrogenation step against W. Linear correlation and logarithmic function are compared.

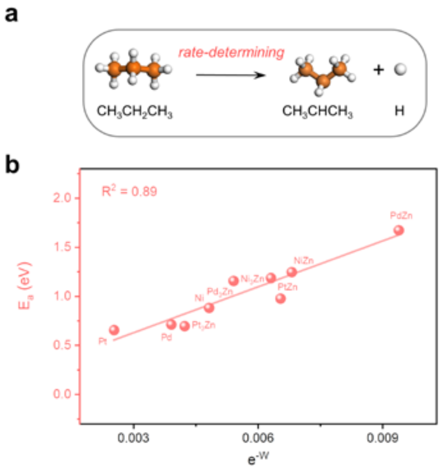

Figure S4. (a) The first C-H activation step of β-type PDH reaction. (b) Plot of the activation energy (E_a) for first dehydrogenation step against e^{-W}.

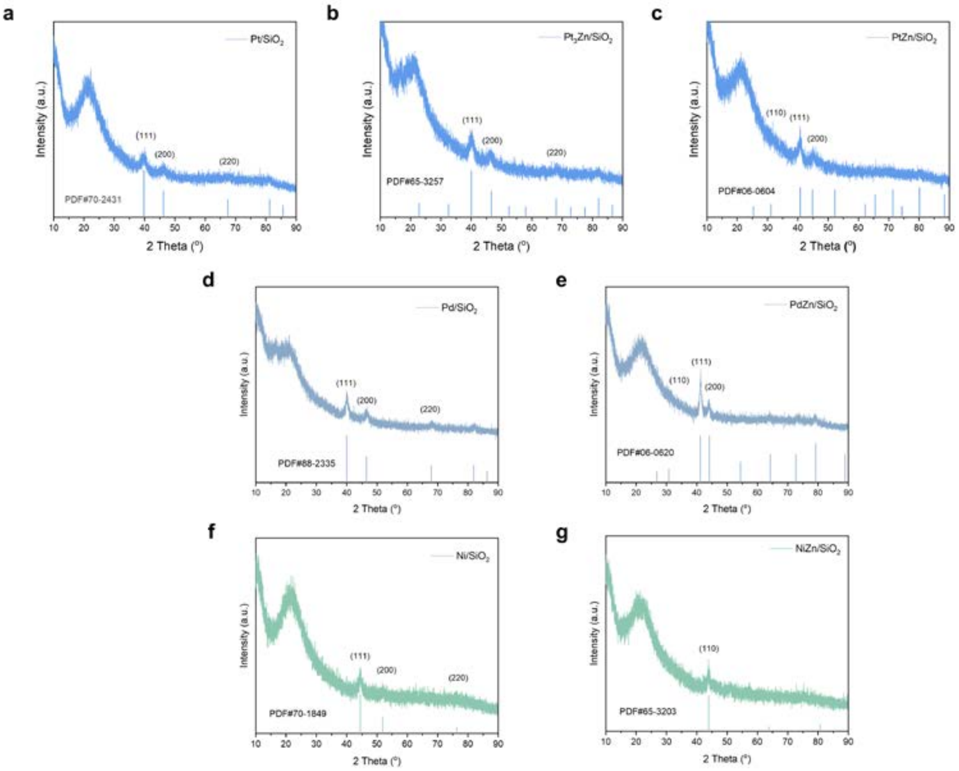

Figure S5. XRD patterns. (a) 2% Pt/SiO_2, (b) 2% Pt_3Zn/SiO_2, (c) 2% $PtZn/SiO_2$, (d) 2% Pd/SiO_2, (e) 2% $PdZn/SiO_2$, (f) 2% Ni/SiO_2, and (g) 2% $NiZn/SiO_2$.

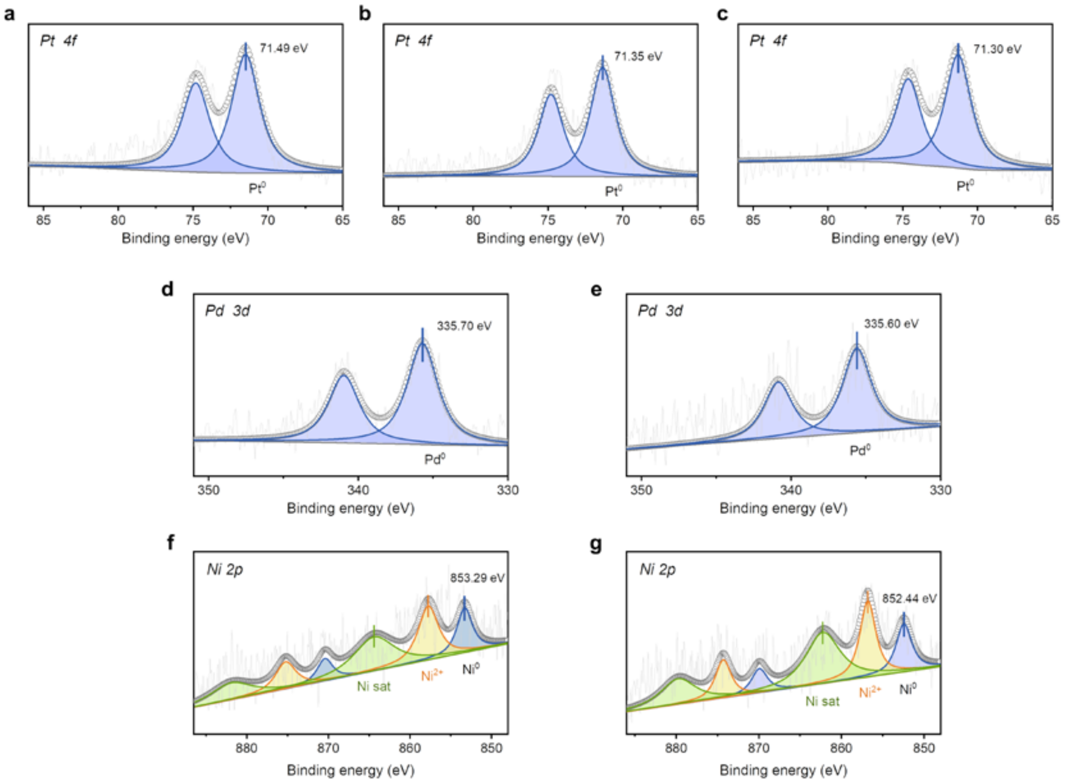

Figure S6. XPS spectra for (a) 2% Pt/SiO_2, (b) 2% Pt_3Zn/SiO_2, (c) 2% PtZn/SiO_2, (d) 2% Pd/SiO_2, (e) 2% PdZn/SiO_2, (f) 2% Ni/SiO_2, and (g) 2% NiZn/SiO_2.

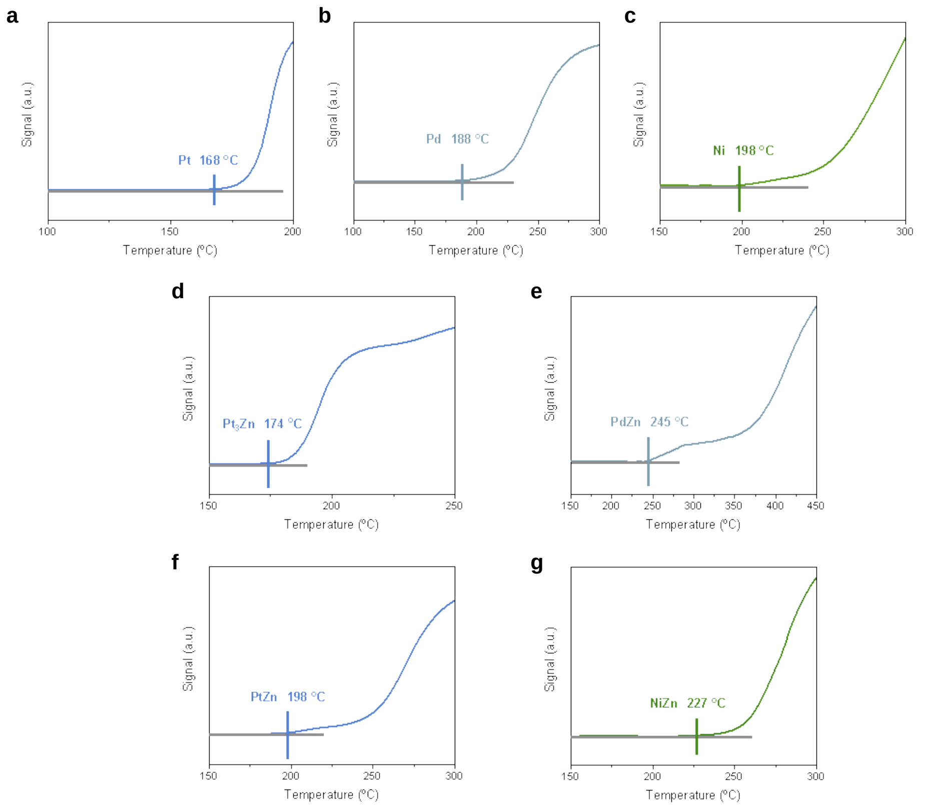

Figure S7. Signals of C_3H_7D during TPSR for P–D scrambling over (a) 2% Pt/SiO_2, (b) 2% Pd/SiO_2, (c) 2% Ni/SiO_2, (d) 2% Pt_3Zn/SiO_2, (e) 2% PdZn/SiO_2, (f) 2% PtZn/SiO_2, and (g) 2% NiZn/SiO_2.

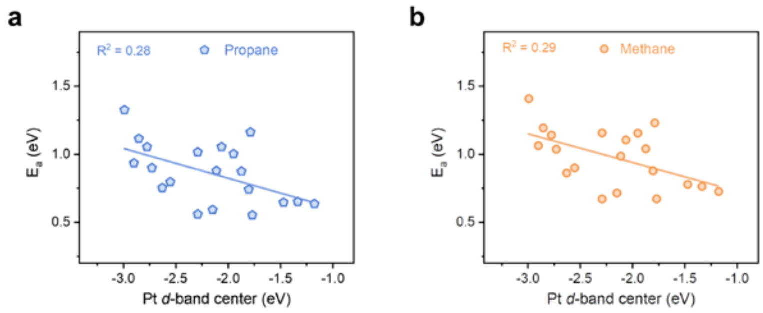

Figure S8. C–H bond activation for different reactants over a series of PtM alloys (M = 3*d*, 4*d*, and 5*d* metals). Plots of the activation energy (E_a) for first dehydrogenation step of (a) propane and (b) methane against the *d*-band center of Pt.

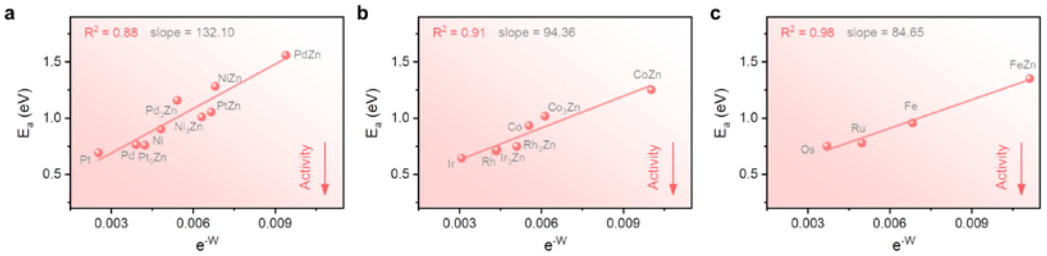

Figure S9. C-H bond activation of propane with metals in group (a) 8, (b) 9, and (c) 10 (M = Fe, Ru, Os, Co, Rh, Ir, Ni, Pd, and Pt) as active sites. Plot of the activation energy (E_a) for first dehydrogenation step against e^{-W}. Not all M_3Zn and MZn are shown here because some structures are unstable and the calculation of transition states is not available.

Table S1. Chemical bonding analyses of M-C bond for M, M_3Zn, and MZn (M = Pt, Pd, and Ni) with top-adsorbed CH_3.

	NBO	Occupancy	Hybridization (%)
Pt-*CH$_3$	Pt-C BD* **(σ*)**	0.26	Pt: s 9, p 3, d 88
	Pt-C BD **(σ)**	1.99	C: s 19, p 81
Pt$_3$Zn-*CH$_3$	Pt-C BD* **(σ*)**	0.32	Pt: s 9, p 4, d 87
	Pt-C BD **(σ)**	1.98	C: s 21, p 79
PtZn-*CH$_3$	Pt-C BD* **(σ*)**	0.46	Pt: s 7, p 4, d 89
	Pt-C BD **(σ)**	1.99	C: s 22, p 78
Pd-*CH$_3$	Pd-C BD* **(σ*)**	0.43	Pd: s 4, p 2, d 94
	Pd-C BD **(σ)**	1.99	C: s 18, p 82
Pd$_3$Zn-*CH$_3$	Pd-C BD* **(σ*)**	0.47	Pd: s 3, p 2, d 95
	Pd-C BD **(σ)**	1.99	C: s 18, p 82
PdZn-*CH$_3$	Pd-C BD* **(σ*)**	0.74	Pd: s 2, p 2, d 96
	Pd-C BD* **(σ)**	1.99	C: s 20, p 80
Ni-*CH$_3$	Ni-C BD* **(σ*)**	0.42	Ni: s 6, p4, d 90
	Ni-C BD **(σ)**	1.98	C: s 22, p 78
Ni$_3$Zn-*CH$_3$	Ni-C BD* **(σ*)**	0.49	Ni: s 5, p 4, d 91
	Ni-C BD **(σ)**	1.97	C: s 24, p 76
NiZn-*CH$_3$	Ni-C BD* **(σ*)**	0.52	Ni: s 6, p 5, d 89
	Ni-C BD **(σ)**	1.97	C: s 24, p 76

Plot-BD*

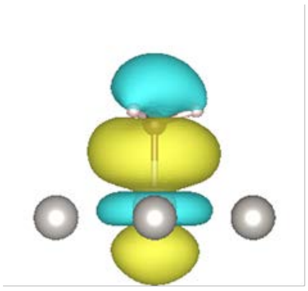

Plot-BD

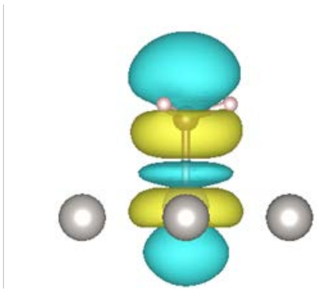

References

1. G. Kresse and J. Furthmüller, *Phys. Rev. B*, 1996, **54**, 11169-11186.
2. G. Kresse and D. Joubert, *Phys. Rev. B: Condens. Matter Mater. Phys.*, 1999, **59**, 1758-1775.
3. J. P. Perdew, K. Burke and M. Ernzerhof, *Phys. Rev. Lett.*, 1996, **77**, 3865-3868.
4. J. Wellendorff, K. T. Lundgaard, A. Møgelhøj, V. Petzold, D. D. Landis, J. K. Nørskov, T. Bligaard and K. W. Jacobsen, *Phys. Rev. B*, 2012, **85**.
5. R. F. W. Bader, *Chem. Rev.*, 1991, **91**, 893-928.
6. G. Henkelman, B. P. Uberuaga and H. Jónsson, *J. Chem. Phys.*, 2000, **113**, 9901-9904.
7. G. Henkelman and H. Jónsson, *J. Chem. Phys.*, 1999, **111**, 7010-7022.
8. S. Maintz, V. L. Deringer, A. L. Tchougreeff and R. Dronskowski, *J. Comput. Chem.*, 2016, **37**, 1030-1035.
9. R. Dronskowski and P. E. Bloechl, *J. Phys. Chem.*, 1993, **97**, 8617-8624.
